# Total hip and knee arthroplasty in HIV- and HCV-positive hemophilia patients: short term follow-up of 14 patients

**DOI:** 10.1186/s12891-023-07087-1

**Published:** 2023-12-06

**Authors:** Zhengming Wang, Yong Gu, Rui Wang, Sicheng Xiang, Zhaokai Jin, Peijian Tong, Shuaijie Lv, Xun Liu

**Affiliations:** 1 Shi’s Center of Orthopedics and Traumatology, Shuguang Hospital, Shanghai University of Traditional Chinese Medicine, Shanghai, China; 2 Translational Medical Innovation Center, Zhangjiagang TCM Hospital Affiliated to Nanjing University of Chinese Medicine, Zhangjiagang, Jiangsu China; 3 Department of Orthopedics, Zhangjiagang TCM Hospital, Nanjing University of Chinese Medicine, Zhangjiagang, Jiangsu China; 4 Guanghua Clinical Medical College, Shanghai University of Traditional Chinese Medicine, Shanghai, China; 5 The First Affiliated Hospital of Zhejiang Chinese Medical University (Zhejiang Provincial Hospital of Chinese Medicine), Hangzhou, Zhejiang China

**Keywords:** Haemophilia, Human immunodeficiency virus, Hip, Knee, Arthroplasty

## Abstract

**Background:**

Haemophilic arthropathy (HA) is a common comorbidity of haemophilia. Some people with haemophilia (PWH) were human immunodeficiency virus (HIV)-positive. Arthroplasty is an effective treatment for end-stage HA. This study was carried out to report the effectiveness and satisfaction following total hip arthroplasty (THA) or total knee arthroplasty (TKA) in PWH with HIV.

**Patients and methods:**

All patients with haemophilia and HIV undergoing THA or TKA in our centre from January 2015 to June 2020 were reviewed. All patients were followed for at least twenty-four months. The improvements in postoperative indicators were evaluated at the latest follow-up, including the Visual Analogue Scale (VAS) score, range of motion (ROM), and validated joint scores such as Knee Society Score (KSS; clinical and functional) and Harris Hip Score (HHS). The complications and satisfaction were analysed likewise. Those were utilized to weigh the risks and benefits of the procedure in the population.

**Results:**

Fourteen patients (7 hips and 14 knees) were included in the study. The follow-up of the THA cohort was 53.3 months (range, 27–82) and the TKA cohort was 50.1 months (range, 25–85), respectively. The average VAS score was ameliorated from 7.3 to 3.0 and 6.6 to 2.8 in the two groups (*P* < .001, respectively). Similarly, two cohorts (THA and TKA) showed statistically significant changes in the extension and flexion ROM between the preoperative and the latest follow-up (*P* < .05, *P* < .001, respectively). Besides, statistically significant differences between the preoperative and final follow-up values of HHS (from 41.6 to 82.3), clinical KSS (from 34.8 to 72.8), and functional KSS (from 42.9 to 73.2) were observed (*P* < .001, respectively). Notably, there were 4 complications noted among 21 arthroplasties performed, giving a 19.0% complication rate. Based on the satisfaction score, the majority of patients were optimistic about the arthroplasty.

**Conclusion:**

Given these findings, THA or TKA of the PWH with HIV is a worthwhile procedure and can be performed by an experienced and collaborative multidisciplinary team in a tertiary centre with a good haemophilia care system.

## Introduction

Haemophilia is a hereditary hemorrhagic disease characterised by the deficiency of one of the coagulation factors, including factor VIII (FVIII) in haemophilia A and factor IX (FIX) in hemophilia B [1]. A prevalence of 29.6 cases per 100,000 males for haemophilia A and B combined with the current world population estimate of 7.5 billion (3.8 billion males) generates an estimated 1,125,000 males with haemophilia [2]. Recurrent spontaneous episodes of bleeding, such as musculoskeletal system bleeding or viscera bleeding, are common in people with haemophilia (PWH), wherein haemorrhage into the former accounts for 80–90% of all [1]. It forms a vicious cycle of haemarthrosis (joint bleeding), leading to friable, hypertrophied synovium, which becomes further prone to bleeding [3]. Moreover, the blood in the joint can result directly in the death of articular chondrocytes. The end-stage haemophilic arthritis (HA) produced by the aforementioned processes destroys the joint and results in debilitating pain, a restricted range of motion (ROM), and changes in functional ability [4, 5]. Supplying factors is very necessary to avoid bleeding and break the cycle, including an infusion of plasma and FVIII or FIX concentrates. However, for various reasons, good management was not given to the PWH in the mid-1980s [6]. Besides, prior to the development of viral inactivation procedures, some of the haemophilic patients who had previously received large pool of plasma-derived factor concentrates were infected with hepatitis C virus (HCV) and human immunodeficiency virus (HIV) [7, 8]. This leads to most PWH suffering from advanced HA and virus infection nowadays.

Total joint arthroplasty (TJA) is the most common intervention for severe haemophilic joint deterioration [9]. With the improvements in pharmacological and surgical interventions, the need for major elective procedures and the opportunity for orthopaedics doctors to encounter such patients in clinical practise have increased [10]. The effectiveness of arthroplasty needs concomitant updating. Consequently, this study was carried out to report outcomes for pain, ROM, functional mobility, complication rates, and satisfaction following total hip arthroplasty (THA) or total knee arthroplasty (TKA) in PWH with HIV during the last 7 years in our centre.

## Materials and methods

### Study design

All patients with haemophilia and HIV who had undergone a THA or TKA in our hospital from January 2015 to June 2020 were reviewed. This retrospective study was approved by the Institutional Ethics Review Board. During the follow-up process, all participants were well informed about this study’s purpose, methods, and procedures, and they were free to withdraw anytime. Inclusion criteria were: (1) a preoperative cluster of differentiation 4 (CD4) count > 200 cells/mm3 and an undetectable HIV load; (2) an end-stage HA of the hip or knee with an Arnold-Hilgartner grade [11] of IV ~ V; and (3) a minimum one-year follow-up. Exclusion criteria were inhibitors, a previous history of hip or knee surgery, an active infection, and a condition that was unable to tolerate arthroplasty. Patients with a poorly controlled HIV or other infection had THA or TKA delayed until they met the criteria.

### Perioperative management

Preoperatively, each patient underwent multidisciplinary treatment (MDT). Apart from some routine radiographic examinations, such as anteroposterior view, lateral view, long-leg standing radiographs, computed tomography, and 3-dimension reconstruction, the lab tests should be given attention. The pharmacokinetics and inhibitors were assessed for every patient preoperatively. According to the combination of the World Federation of Haemophilia guidelines [12] and our experience, FVIII substitution therapy was infused, aiming for a factor level of 80–100% at the time of surgery. The level above 60% was maintained by continuous factor infusion in the immediate postoperative period (from day 1 to 3) and then 40–60% in the late postoperative period (from day 4 to 6). A factor level of above 30% was achieved from post-op 7–14. Perioperative safe ranges of FIX levels were maintained similarly to FVIII. An extra factor dose should be administered if necessary. The arthroplasty will be scheduled based on a CD4 count, an undetectable viral load, and the pharmacokinetic effect.

### Total hip and knee arthroplasty

**Fig. 1:**
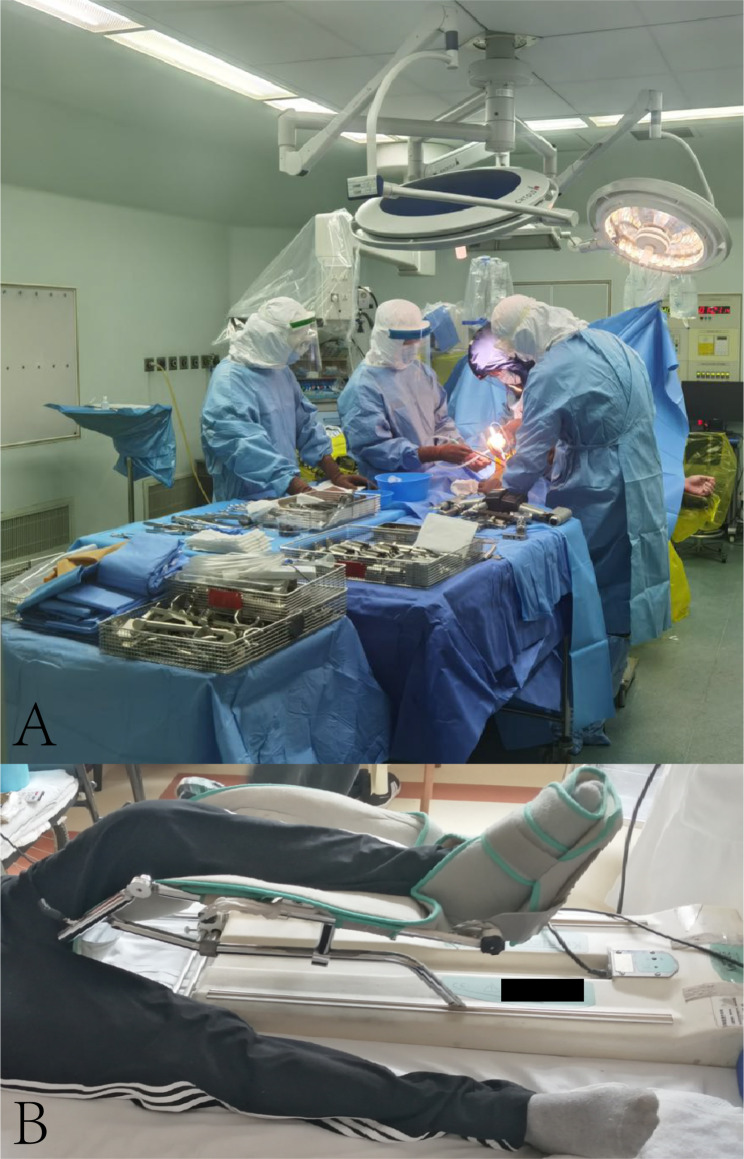
Total knee arthroplasty and postoperative rehabilitation. **(A)** Surgeons with protective equipment in the operating room; **(B)** Postoperative rehabilitation with a continuous passive motion (CPM) machine in one case

All patients received prophylactic antibiotics of cefuroxime within 30 min preoperatively and 3–5 days postoperatively, and general anaesthesia was used in all THAs and TKAs. All the procedures were performed by the same experienced surgeon team with tranexamic acid under complete protection in a hundred-level laminar flow operating room (Fig. 1A).

Every patient in the THA cohort underwent the posterolateral approach. Hemostasis was rigorously preserved throughout the procedure, while proliferative synovial tissues were completely removed. The removal of proliferative osteophytes allowed for the appropriate release of muscle contracture or fibrosis surrounding the joint capsule and the restoration of the joint’s range of motion [13]. Typically, vacuum drainage was employed and withdrawn 12 to 24 h after surgery. Cementless implants with ceramic-on-ceramic surface bearings were used to treat all patients. On the other side, the pneumatic tourniquet was inflated before the incision and deflated after the closure for TKAs. The arthroplasty used a midline anterior incision with a medial parapatellar approach. According to the standard surgical techniques, a fully osteotomy, releasing the inner and outer soft tissues and the posterior joint capsule, and removing synovium completely were administered. There was no drainage in any patient. All femoral and tibial components were fixed in place using cement with antibiotics.

During the operation, if massive bleeding occurred, patients were immediately given a transfusion and an additional factor. We have not prescribed any drug as thromboembolic prophylaxis.

### Postoperative rehabilitation

Weight-bearing with crutches was allowed as tolerated on the next day of surgery, similar to non-haemophiliac patients. Furthermore, under the guidance of the rehabilitation physician, active and passive rehabilitation training began after coagulation factor replacement therapy (Fig. 1B). It was crucial to keep an eye on factor concentrations and joint haemorrhage during rehabilitation, which was promptly suspended, and the RICE (rest, ice, compression, and elevation) approach was followed whenever a bleeding episode happened [13]. Keep the incision dry and clean, and always pay attention to whether infection occurs.

### Outcomes evaluation

The visual analogue scale (VAS) score and ROM were used to evaluate pain and improvement of joint motion. The extension ROM (i.e., flexion contracture) demonstrated the changes towards full extension (degree), and the flexion ROM demonstrated the changes of the hip or knee arc. Besides, validated joint scores were utilised in the assessment of each joint: clinical and functional Knee Society Score (KSS) for knees [14] and Harris Hip Score (HHS) for hips, both preoperatively and at the lastest follow-up [15].

Complications were recorded, such as incision complications, infections, joint bleeding, thrombosis, and prosthesis loosening. X-rays were scrutinised for any sign of loosening. A change in the position of the component or progressive radiolucent lines was considered a radiologic failure. Patients’ satisfaction consisted of satisfaction with pain relief, satisfaction with functional improvement, and overall satisfaction with treatment procedures, which were performed respectively with a number from 1 to 100 in the latest follow-up. The aforementioned indicators were assessed by the independent reviewer.

### Statistical analysis

All data analyses were performed using SPSS version 25.0 (IBM, Armonk, NY, USA). The mean ± standard deviation, median (IQR), or frequency (percentage) was used to summarise variables as appropriate. The Shapiro-Wilk test was applied to test the normality of the data. The preoperative clinical results were compared to the postoperative results (paired t test). A *p* value of < 0.05 was considered statistically significant.

## Results

### Demographics

The summary of patients’ detailed demographics is shown in Table 1. Five patients (7 hips) and nine patients (14 knees) were included in the study. All of the patients were male. There were a total of fourteen patients, including 12 patients with haemophilia A and two haemophilia B cases. In the THA cohort, the median age of the patients was 30 [9] years, with a range of 23 to 56 years, and their mean BMI was 22.5 ± 2.0 kg/m^2^. However, in the TKA cohort, the mean age of the patients was 38.9 ± 8.4 years, with a range of 20 to 53 years, and the median BMI of them was 21.1 (2.2) kg/m^2^. The mean duration of clinical follow-up was 53.3 months (with a range of 27 to 82 months) and 50.1 months (with a range of 25 to 85 months), respectively. Among the patients, one had mild haemophilia, 7 had moderate haemophilia, and 9 had severe haemophilia. There were CD4 counts in each patient in excess of 200 cells/mm^3^. All patients in both groups were co-infected by HIV and HCV, while one patient in each cohort was infected with the hepatitis B virus. The operation time and length of hospital stay (LOS) were listed in Table 1. No patient was lost to follow-up, and there were no deaths during the study period.

**Table 1:** Summary of demographics

Demographic and Surgical Data	THA	TKA
Male (*n* / %)	5/100%	9/100%
Age (years)	30.0 (9.0)	38.9 ± 8.4
BMI (kg/m^2^)	22.5 ± 2.0	21.1 (2.2)
Arthroplasty Count (*n*)	7	14
Factor VIII Deficiency (*n* / %)	5/100%	7/77.8%
Hemophilia Severity (*n* / %)		
Mild	0	1/7.1%
Moderate	3/42.9%	4/28.6%
Severe	4/57.1%	9/64.3%
CD4 Count (cells/mm^3)^	520.6 ± 83.9	504.8 ± 121.7
Coinfections (*n* / %)		
HBV	1/20.0%	1/11.1%
HCV	5/100%	9/100%
Operation of Left Side (*n* / %)	2/28.6%	5/35.7%
Operation Time (min)	137.1 ± 10.5	133.4 ± 15.7
LOS (days)	23.1 ± 5.8	25.9 ± 10.8
Follow-up (months)	53.3 ± 20.2	50.1 ± 19.5
Months of follow-up; range	27–82	25–85

THA, total hip arthroplasty; TKA, total knee arthroplasty; BMI, body mass index; CD4, cluster of differentiation 4; HBV, hepatitis B virus; HCV, hepatitis C virus; LOS, length of hospital stay

### Clinical outcome

The average VAS score was ameliorated from 7.3 to 3.0 in the THA group (*P* < .001, Table 2). Similarly, the TKA group had an improvement in VAS score from 6.6 to 2.8 compared with the preoperative group (*P* < .001). Both groups (THA and TKA) showed statistically significant changes in flexion contracture and flexion ROM (*P* < .05, respectively). The THA group (from 13.6° to 0, *P* < .05) and the TKA group (from 18.5° to 6.9°, *P* < .001) showed statistically significant differences in the extension ROM (flexion contracture) between the preoperative and the latest follow-up. The THA groups (from 82.1° to 102.9°) and the TKA groups (from 63.9° to 105.0°) showed statistically significant changes in the flexion ROM (*P* < .001, respectively).

**Table 2:** Comparison of clinical outcomes preoperatively and the latest follow-up

	Preoperative	End of follow-up	*P* value
VAS (points)			
Hip	7.3 ± 0.9	3.0 ± 1.1	< 0.001*
Knee	6.6 ± 0.8	2.8 ± 0.8	< 0.001*
ROM (°)			
Extension ROM (flexion contracture)			
Hip	13.6 ± 6.9	0 (5.0)	0.017*
Knee	18.5 ± 7.5	6.9 ± 5.8	< 0.001*
Flexion ROM			
Hip	82.1 ± 14.4	102.9 ± 7.0	< 0.001*
Knee	63.9 ± 17.6	105.0 (8.5)	< 0.001*
HHS (points)	41.6 ± 10.6 (range: 26–57)	82.3 ± 6.5 (range: 72–91)	< 0.001*
KSS (points)			
Clinical KSS	34.8 ± 10.1 (range: 17–54)	72.8 ± 8.0 (range: 61–92)	< 0.001*
Functional KSS	42.9 ± 13.8 (range: 15–70)	73.2 ± 9.5 (range: 60–90)	< 0.001*

VAS, visual analog scale; ROM, range of motion; HHS, Harris Hip Score; KSS, Knee Society Score. * indicates *P* value < 0.05

**Fig. 2:**
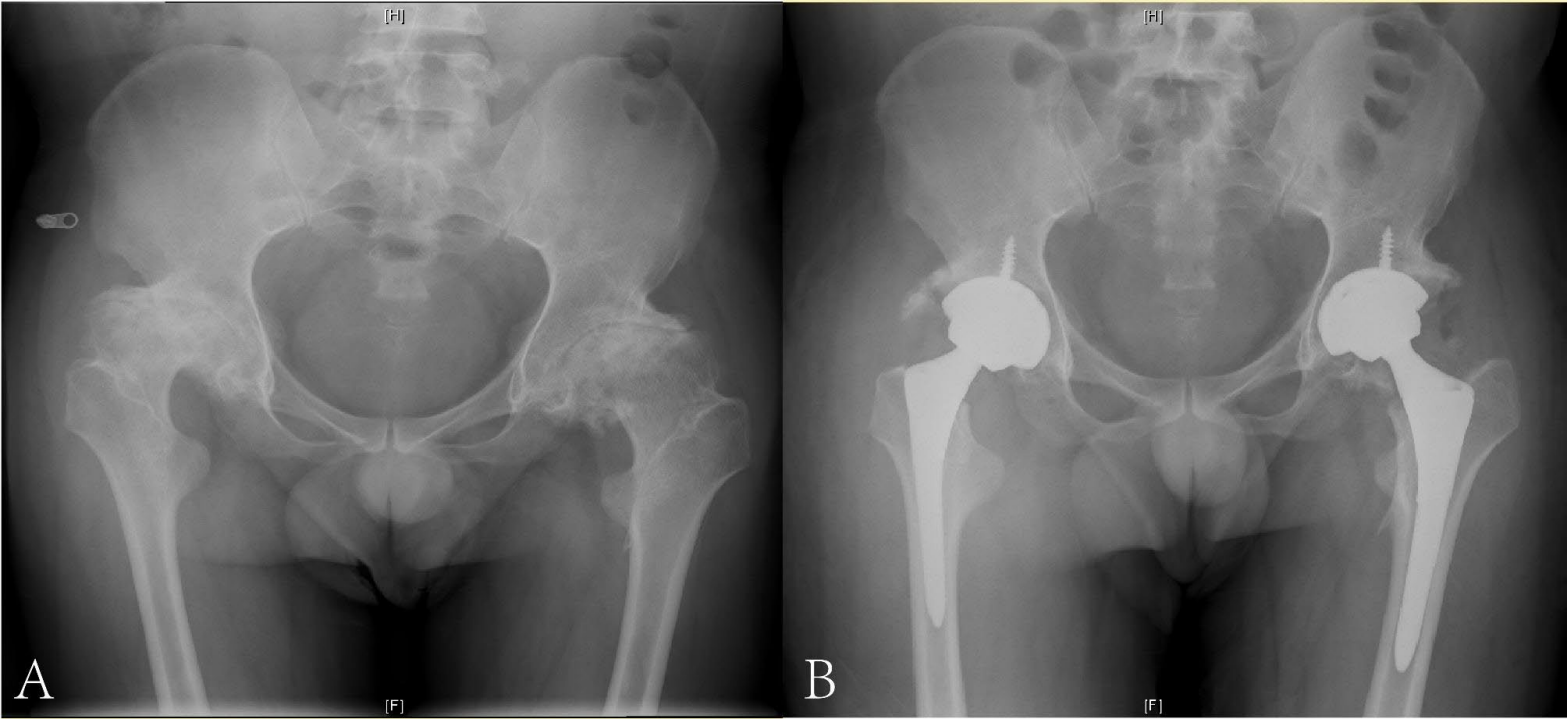
Radiographs of a male patient with advanced bilateral haemophilic hip arthropathy. **(A)** Preoperative anteroposterior view. **(B)** The latest follow up anteroposterior radiograph after total hip arthroplasty twice

**Fig. 3:**
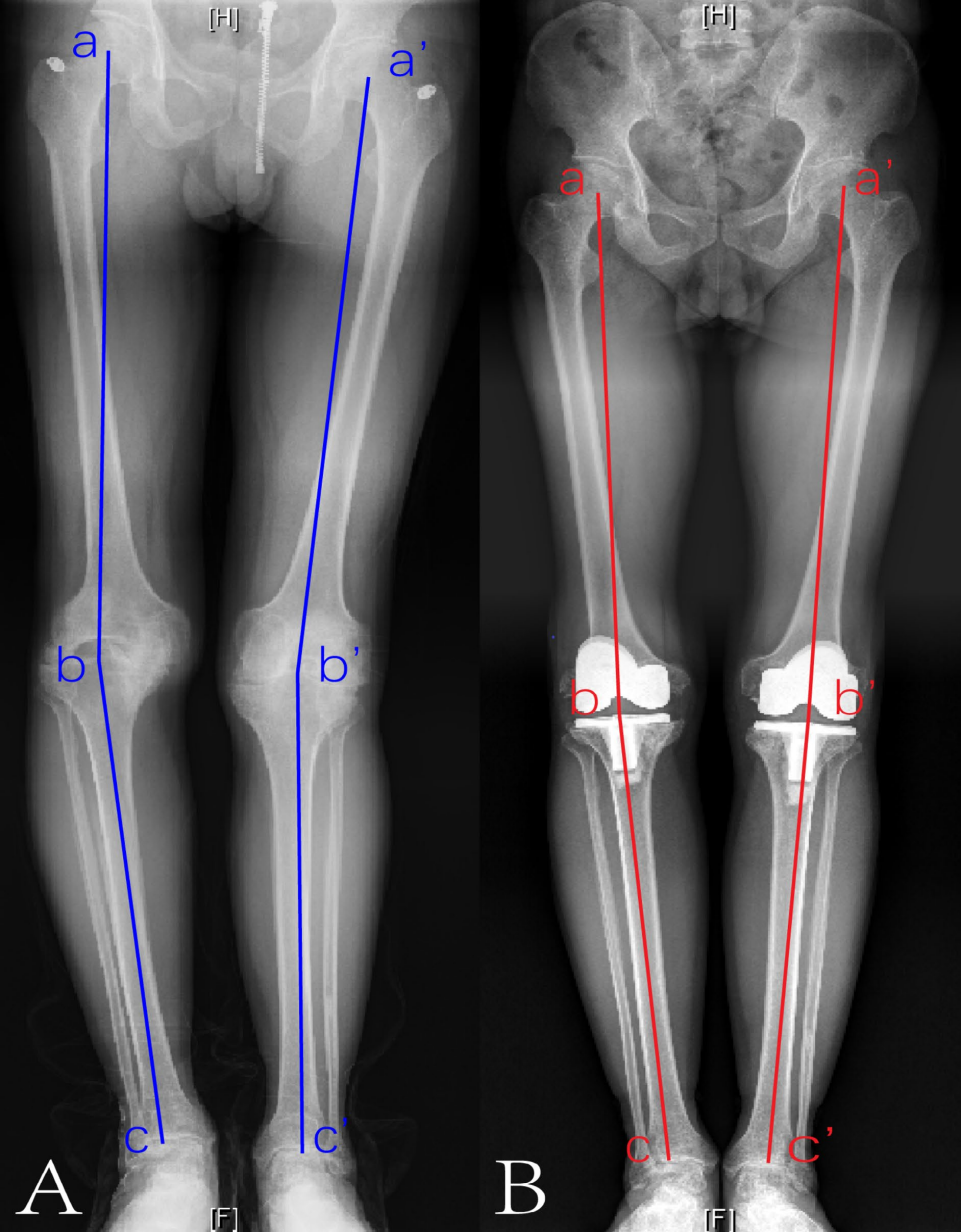
Long-leg standing radiographs of a male patient with advanced bilateral haemophilic knee arthropathy. **(A)** Preoperative anteroposterior view. Blue line: the preoperative mechanical axis. (a and a’: the centre of the right and left femoral head, respectively; b and b’: the centre of the right and left knee, respectively; c and c’: the centre of the right and left ankle, respectively.) **(B)** The latest follow up anteroposterior radiograph after total knee arthroplasty twice. Red line: the postoperative mechanical axis. (a and a’: the centre of the right and left femoral head, respectively; b and b’: the centre of the right and left knee, respectively; c and c’: the centre of the right and left ankle, respectively.)

The mean HHS was 82.3 (range, 72–91) in the latest follow-up (Table 2). Based on the HHS classifications, results were excellent (≥ 90 points) in one hip, good (80–89 points) in four hips, and fair (70–79 points) in two hips. The radiographs of the preoperative and the latest follow-up of a case are illustrated in Fig. 2. There were significant differences between the preoperative and final follow-up values of clinical KSS and functional KSS (*P* < .001, respectively), as shown in Table 2. The mean clinical KSS was 72.8 (range, 61–92). Based on the KSS classifications, results were excellent (≥ 80 points) in three knees, good (70–79 points) in six knees, and fair (60–69 points) in five knees. The functional KSS was higher than the clinical score, with a mean of 73.2 points (range, 60–90). Likewise, the long-leg standing radiographs of the preoperative and the latest follow-up of a case are shown in Fig. 3.

### Complications and satisfaction

There were 4 complications noted among 21 arthroplasties performed, giving a 19.0% complication rate (Table 3). Although the factor was infused as planned, one patient with haemophilia A developed subcutaneous bleeding at the incision after the procedure for five days, which could be attributable to the high-intensity rehabilitation exercise in light of the patient’s statement. He was managed by reducing the intensity of rehabilitation exercise and immediately injecting 1000 U of factor VIII. The main postoperative complications were incision-poor healing in the two cohorts, which might be related to the nature of patients with HIV. There was no infection, deep vein thrombosis, or other adverse events in the study.

**Table 3:** Complication and satisfaction in two cohorts

	THA	TKA	Total
Complications (*n* / %)			
Bleeding	0	1/7.1%	
Poor incision healing	1/14.3%	2/14.3%	
DVT	0	0	
Prosthesis loosening	0	0	
Periprosthetic fracture	0	0	
Infection	0	0	
Surgical site infection	0	0	
Periprosthetic joint infection	0	0	
Total	1/14.3%	3/14.3%	4/19.0%
Satisfaction (points)			
Pain	89.3 ± 6.1	83.9 ± 7.4	85.7 ± 7.3
Joint function	80.7 ± 6.7	81.8 ± 8.2	81.4 ± 7.6
Whole treatment procedure	87.9 ± 5.7	89.6 ± 7.2	88.6 ± 7.1

THA, total hip arthroplasty; TKA, total knee arthroplasty; DVT, deep vein thrombosis

All patients’ satisfaction with pain relief, functional improvement, and the whole treatment process were scored as 85.7 points, 81.4 points, and 88.6 points, respectively, at the end of the follow-up (Table 3). Moreover, satisfaction with pain relief was higher than functional improvement in the two groups. Based on the satisfaction score, the majority of patients were optimistic about the arthroplasty.

## Discussion

In view of repeated articular bleeding episodes from early childhood, PWH commonly has a severe degree of joint damage by the time they reach adulthood. Despite the widespread availability of factor replacement nowadays, patients continue to be at risk of developing chronic HA [16]. It is well known that TJA is an effective, safe, and well-established procedure for the treatment of terminal osteoarthritis [17–19]. However, TJA for HA with HIV is technically demanding because of anatomical distortion, soft tissue fibrosis, flexion contractures, and poor bone quality [6]. In addition, perioperative management is complicated, which consists of the regulation of factor concentration, protection of both staff at the hospital and the patient, CD4 count, and viral load. Further complications arose in the case of PWH who were also HIV positive with CD4 counts lower than 200 cells/mm^3^ in particular [20, 21]. With the risks of postoperative infection in the HIV-positive haemophilic patient and the high rate of unsatisfactory results that have been reported, several studies [22, 23] disapproved of major elective procedures in this population. Nevertheless, given its amazing effectiveness in relieving pain and improving function and quality of life, some controversies [24–26] exist as to whether the benefits of TJA outweigh the reported disadvantages in these patients. Moreover, due to the advances in perioperative care and implants and some encouraging reports [16, 26–30] of arthroplasty in HIV in recent years, the balance is turning gradually. There is a rising need to better understand the role of TJA among PWH with HIV patients. Accordingly, it is imperative to analyse and demonstrate the outcomes of this procedure to establish its effect on this patient population.

Our study found that THA or TKA in PWH with HIV was successful in treating HA, as previously evidenced by an improvement in HHS, Hospital for Special Surgery Knee Score (HSSKS), and KSS (clinical and functional) in reported studies [16, 29, 31]. The outcomes of THA and TKA in these patients were comparable to those of haemophiliacs in terms of pain relief and ROM improvement [28, 29]. It has been confirmed that the operation may give most patients years of life without pain. Besides, flexion contractures have been demonstrated to increase energy expenditure, force on the contralateral lower extremity, and reduce walking velocity [32, 33]. Furthermore, Ritter et al. [34] showed that patients with a residual flexion contracture of more than 10° had worse pain scores and function scores than those who achieved full extension. Taking the results of the above studies into account, we recommend that arthroplasty be performed as early as possible in patients who meet the criteria and conditions for surgery and that the deformity be rectified as far as possible during the procedure. For our study, the postoperative flexion contractures were 0° and 6.9° in THA and TKA, respectively.

There were not without challenges performing THA and TKA in the population, one of which is a complication. Many factors could play a role in the decreased complication rate, such as the use of highly active antiretroviral therapy, the use of antibiotic-loaded cement, sufficient factor replacement preoperatively, and prophylactic antibiotics prior to invasive procedures [35]. However, there was a 19.0% complication rate under meticulous care in our study. The main postoperative complications were incision poor healing in the 21 arthroplasties, which suggested that more attention should be attached to the incision healing condition and that suture removal may need to be delayed in the PWH with HIV. Additionally, because of the high-intensity rehabilitation exercise, one patient given enough factors developed subcutaneous bleeding at the incision. It is necessary to educate and guide patients to carry out planned rehabilitation. Astonishingly, no infection was detected in our study, which could be owed to the CD4 count greater than 200 cells/mm^3^, the undetectable viral load, and well-comprehensive perioperative management. This was consistent with the results of Graham et al. [36]. And one finding during the operation was osteoporosis in these patients. It is well noted that patients with haemopilia or HIV tend to have decreased bone mineral density compared with negative individuals [37, 38]. It has been hypothesized that the continuous activation of some molecules creates a chronic indolent inflammatory cascade, disrupting the balance of bone resorption and formation by stimulating osteoclasts to resorb the bone [39, 40]. It remained to be further researched.

The satisfaction score was higher than 80 points in pain relief, functional improvement, and the whole treatment process, respectively. The satisfaction disparity can be attributed to residual symptoms and deformity, compared with the reported arthroplasty satisfaction for advanced osteoarthritis [41]. Remarkably, the involvement of other joints by HA would affect the functional outcome of THA or TKA, which could be a potential reason that satisfaction with functional improvement was lower than pain relief in the two groups. It is speculated that because the hip has more mobile functions, the satisfaction score of joint function in the TKA group (81.8 ± 8.2 points) was higher than the THA group (80.7 ± 6.7 points) in our study. All patients’ satisfaction with the whole treatment process scored 88.6 points at the end of the follow-up. Combined with the complication rate, despite some disadvantages, THA or TKA is a worthwhile procedure for severe HA with HIV.

Unquestionably, there are some limitations to our study. To begin with, the principal limitation is the nature of the retrospective work, which might be more susceptible to selection bias and confounding. Our results need to be demonstrated by high-quality research. Additionally, on account of the rarity of haemophilia, the sample size was relatively small, and the control group did not exist. The absence of a control group of TJA patients with haemophilia not infected by HIV is another deficit of this study. Then, this is a single-center study, and its results may not be applicable to all centers. A prospective, controlled, multi-center, and large sample study with a large sample may be conducive to the above problem. Nevertheless, our study explored the effects and complications of THA and TKA in the PWH with HIV, which is of significance to the field of arthroplasty.

## Conclusion

After a comprehensive assessment, THA and TKA of the PWH with HIV can be performed by an experienced and collaborative team in a medical center with a good haemophilia care system, which improves joint function and ROM and relieve pain. And the majority of patients were satisfied with the procedure.

## Data Availability

The data used and analysed by this study are available from the corresponding author on reasonable request.

## Abbreviations

FVIII: factor VIII
FIX: factor IX
PWH: people with haemophilia
HA: haemophilic arthritis
ROM: range of motion
HCV: hepatitis C virus
HIV: Human Immunodeficiency Virus
TJA: total joint arthroplasty
THA: total hip arthroplasty
TKA: total knee arthroplasty
CD4: cluster of differentiation 4
MDT: multi-disciplinary treatment
VAS: visual analogue scale
KSS: Knee Society Score
HHS: Harris Hip Score
LOS: length of hospital stay
